# Electrophysiological recording of hypothalamic brain regions in vivo using the transpharyngeal surgical approach in the rat

**DOI:** 10.1111/jne.70177

**Published:** 2026-04-01

**Authors:** Mike Ludwig, Gareth Leng, Colin H. Brown

**Affiliations:** ^1^ Institute for Neuroscience and Cardiovascular Research University of Edinburgh Medical School Edinburgh UK; ^2^ Centre for Neuroendocrinology, Department of Physiology, Faculty of Biomedical Sciences University of Otago Dunedin New Zealand

**Keywords:** hypothalamus, microdialysis, oxytocin, supraoptic nucleus, vasopressin

## Abstract

Transpharyngeal (ventral) surgery in urethane‐anaesthetised rats allows in vivo electrophysiological recording and/or imaging from superficial hypothalamic brain regions and from the pituitary gland. This surgical approach leaves the whole brain intact, providing a stable platform to study single or multiple identified cells over several hours with all central and peripheral inputs intact and the endocrine system functioning, which allows repeated drug application and stimulation of afferent inputs, as has been done for the arcuate nucleus, organum vasculosum of the lamina terminalis (OVLT) and suprachiasmatic nucleus (SCN) inputs to the supraoptic nucleus (SON). Exposing the ventral surface of the brain also allows simultaneous microdialysis for drug administration directly into the SON and for the collection of dialysate samples for measurement of somatodendritic neuropeptide release without disruption of the brain parenchyma. The most recent development using transpharyngeal surgery is two‐photon imaging from the vasculature of the ventral surface of the brain, which has given insight into how SON neuronal activity affects cerebral blood flow and has identified a previously unknown SCN to OVLT portal blood system. Here we provide a brief history of the development of transpharyngeal surgery in the rat, instructions to complete the surgery and suggestions for future studies, extrapolating from the most recent developments in its use.

## 1 INTRODUCTION

The supraoptic nucleus contains the cell bodies of oxytocin and vasopressin neurones that secrete their respective hormones into the bloodstream from their axon terminals in the posterior pituitary gland and their dendrites into the brain.^1^ Vasopressin regulates body fluid balance by increasing renal water reabsorption in response to increasing plasma osmolality.^2^ While the principal hormonal roles of oxytocin are to stimulate uterine contraction during parturition and milk ejection during suckling, oxytocin also contributes to body fluid regulation by increasing natriuresis in response to increasing plasma osmolality, at least in the rat.^3^ However, the basal plasma concentrations of both oxytocin and vasopressin are very low (1–10 pg/mL), making frequent sampling impractical, so our current understanding of the regulation of secretion has depended less on measuring these hormones in the blood than on observing the electrical activity of oxytocin and vasopressin neurones in vivo. That has critically depended on the ability to accurately identify these neurones as they are being recorded.

## 2 DEVELOPMENT OF TRANSPHARYNGEAL SURGERY IN THE RAT

While ex vivo preparations allow investigation of mechanisms that underpin neuronal activity, these techniques do not alone provide great insight into the functional impact of each mechanism on the activity of the neurones in vivo. Such insights are best provided by monitoring neuronal activity in the intact animal, which is increasingly being used in chronically instrumented, freely‐behaving rodents. While such experiments are extremely valuable, they can have limited utility in determining the central regulation of peripheral physiology because it would be unethical to use the required manipulations in conscious animals. Hence there is still a need for electrophysiological recording from anaesthetised animals to connect central mechanisms with peripheral physiology.

Stereotaxic electrode placement is used for most in vivo electrophysiological recordings from the brains of anaesthetised animals. While this approach can also be used to record from the hypothalamus, the relatively large distance from the dorsal surface of the brain to the hypothalamus and the relatively small targets within the hypothalamus make the dorsal approach using stereotaxic techniques challenging. In addition, the dorsal approach also compromises the ability to place other apparatus in the same brain region as the recording electrode. These issues led to the development of the transpharyngeal (ventral) surgery to access and manipulate the hypothalamic nuclei in the anaesthetised rat. The transpharyngeal approach was particularly attractive for the supraoptic nucleus because this brain area lies on the ventral surface of the brain, lateral to the optic chiasm, and so was amenable to direct manipulation during electrophysiological recording. Inspired by Eric Kandel's direct exposure of the preoptic area and pituitary gland in the goldfish,^4^ Yagi et al. were, to our knowledge, the first to use the transpharyngeal approach in anesthetised rats.^5^ Although they did not describe the surgery in detail, they exposed the ventral surface of the hypothalamus to place a recording electrode in the supraoptic nucleus and a stimulating electrode on the neural stalk. Their experiments established that the axons of neurosecretory neurones of the supraoptic nucleus conducted action potentials and introduced the critically important technique of “antidromic identification” to studies of magnocellular neurones. This technique rests on the fact that electrical stimuli applied to the neural stalk will trigger action potentials (spikes) in the axons of magnocellular neurones. These spikes travel both ‘orthodromically’ to the nerve terminals in the posterior pituitary, but also ‘antidromically’ to the cell bodies in the supraoptic and paraventricular nuclei. The antidromic spikes will follow every stimulus in a short train of stimuli presented at 50–100 Hz, and each antidromic spike will reach each neuronal cell body at a fixed latency that depends on the axon length and conduction velocity. However, if a spontaneous spike occurs just before a stimulus is applied, then the orthodromically conducted spontaneous spike will meet the antidromic spike along the axon, and both will be extinguished by the collision. Demonstrating these phenomena (‘constant latency’, ‘frequency following’ and ‘collision’) for each neuron became standard tests for ‘antidromic identification’, accepted as rigorous evidence that a neurone recorded from the region of the supraoptic nucleus projected to the neural lobe.

Within a few years after the publication of Yagi's paper, virtually all electrophysiological studies of magnocellular neurones in vivo used antidromic identification. Most of these studies used a stereotactic approach to the supraoptic or paraventricular nucleus and recorded in lactating rats where a stimulating electrode could be accurately placed on the neural stalk by observation of the intramammary pressure response to stimulation.^6^, ^7^ However, Dreifuss and Kelly described a transpharyngeal surgical approach to record from the supraoptic nucleus,^8^ and their approach was subsequently modified by Leng, who first used it to test the intrinsic osmosensitivity of supraoptic neurones by using a ‘microtap’ to apply tiny amounts of hypertonic saline directly to the surface of recorded neurones.^9^

In parallel with the development of transpharyngeal surgery for electrical recording from the supraoptic nucleus, a transpharyngeal approach was also developed for gonadotrophin releasing hormone (GnRH) sampling from neurohypophysial portal blood,^10^ similar to the procedure for exposure of the neural stalk described in the next section. Later, a different ventral approach was developed in the mouse for pituitary growth hormone cell visualisation,^11^ which was adapted for hypothalamic GnRH neurone visualisation.^12^

## 3 URETHANE ANAESTHESIA

Transpharyngeal surgery is used to study single or multiple identified cells over several hours. Therefore, steady long‐term anaesthesia is required to provide a stable platform for experimental manipulations. Urethane (ethyl carbamate) is the anaesthetic of choice because it induces long‐lasting anaesthesia by the summation of modest effects on many neurotransmitter systems and has minimal impact on autonomic and cardiovascular systems.^13^

Unlike other anaesthetics, urethane does not appear to impair neuroendocrine reflexes, with no sustained effect on the spontaneous activity of hypothalamic neurones.^14^, ^15^ A direct comparison between the electrical activity of neurones in conscious and anaesthetised rats was made by Summerlee and Lincoln, who showed that the electrophysiological characteristics of oxytocin neurones in lactating rats and their responses to suckling are indistinguishable from those reported from urethane‐anaesthetised rats.^15^ We have recently discussed in detail the issues of using urethane for recordings of supraoptic vasopressin and oxytocin neurones.^16^

A single intraperitoneal injection of urethane (1.25–1.50 g/kg) is sufficient to keep rats surgically anesthetised for over 12 h. External warming is not applied because failure of the hypothalamus to maintain body temperature suggests that other hypothalamic functions are probably also compromised. Nevertheless, rats are wrapped in insulating material to reduce heat loss.

Before commencing transpharyngeal surgery, standard surgical techniques are used to catheterise the trachea, which maintains a patent airway during the surgery and subsequent manipulations. Other catheters can be inserted as needed: in the femoral vein and/or a branch of the jugular vein for drug administration; in the femoral artery to monitor blood pressure or withdraw blood samples; in the cerebral ventricles for central drug administration. The vascular catheters should be filled with isotonic saline; heparin should not be used until after surgery is complete because the lack of coagulation can be problematic. Small pieces of absorbable haemostat can be used to staunch any bleeding that enters the surgical exposure.

## 4 THE TRANSPHARYNGEAL SURGERY IN THE RAT—A STEP‐BY‐STEP GUIDE

**FIGURE 1:**
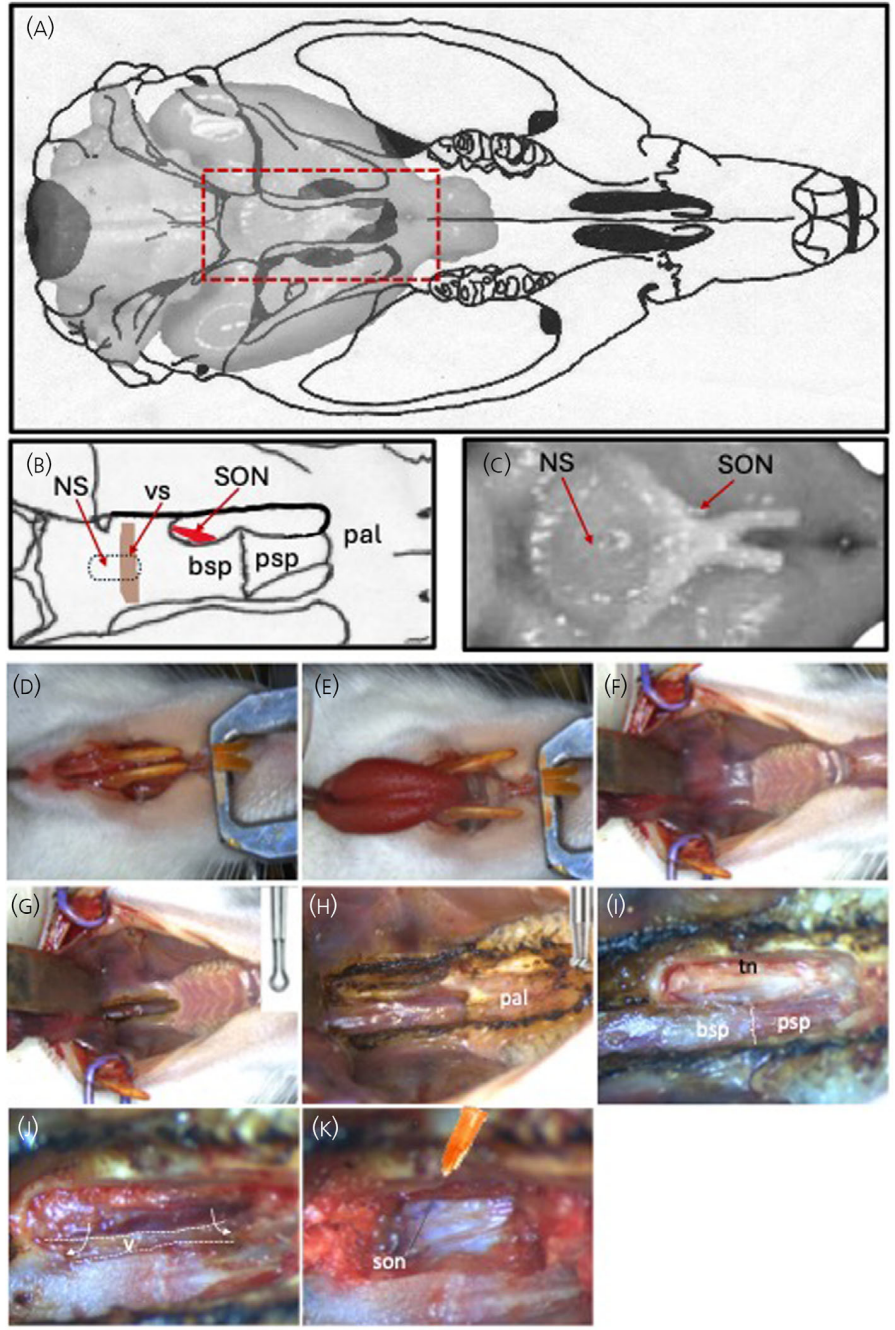
Step‐by‐step guide to transpharyngeal exposure of the supraoptic nucleus. (A) Schematic representation of the rat brain within the skull. (B) and (C) Enlarged inset from (A) showing important bone structures (B) and the location of the supraoptic nucleus and neural stalk in the brain (C). (D) The rat is placed supine in a head holder using ear, incisor and nose bars. (E) The ranii of the mandible are separated with scissors and the tongue is pulled between the lower incisors, and held in place with a retractor. (F) Two wire retractors are used to retract the lower incisors laterally. (G) The soft palate is cauterized with a fine electrocautery knife along its visible extent on the midline. The cautery is extended bilaterally to the medial aspect of the left and right zygomatic arches. (H) The rugae overlying the hard palate (pal) are cauterised and the hard palate is removed using a drill. (I) The presphenoid bone with the suture between the presphenoid (psp) and basisphenoid (bsp) bones will now be visible. To expose the supraoptic nucleus, the lateral wing of the palatine bone and medial pterygoid plate are cleared of muscle using cautery. The exposed bone is drilled away to expose the trigeminal nerve (tn) bundle. (J) The nerve is removed from above the supraoptic nucleus using two pairs of fine forceps, leaving the supraoptic nucleus obscured by a large vein. (K) The vein is compressed with pledgets of paper tissue that are wedged against the sphenoid bones rostrally and caudally. A fine needle is used to make a small incision through the meninges to allow the recording electrode access to the supraoptic nucleus. The recording electrode is best placed as close to the bifurcation of the communicating artery and the middle cerebral artery, next to the optic chiasm. bsp, basisphenoid; NS, neural stalk; pal, palate; psp, presphenoid; SON, supraoptic nucleus; tn, trigeminal nerve; v, vein; vs, venous sinus.

The rat is then placed supine in a head holder using standard ear, incisor and nose bars (but with the incisor/nose bar assembly upside down in the frame) on an anti‐vibration table with the upper incisors raised ~5 mm above the interaural line. The ranii of the mandible are separated with scissors, avoiding cutting either the skin of the lower jaw or the tongue, and the tongue is pulled between the lower incisors and held in place with a retractor. Two wire retractors are used to retract the lower incisors laterally, “gaping” the mouth, revealing the soft palate and hard palate (Figure 1D–F).

Under a dissecting microscope with a long focal length objective, the soft palate is cauterized with a fine electrocautery knife along its visible extent on the midline. The cautery is then extended bilaterally to the medial aspect of the left and right zygomatic arches. The rugae overlying the hard palate are then cauterised and the hard palate is removed using a high‐speed drill with a 1‐mm burr. The nasal membrane overlying the sphenoid bones is removed using fine forceps. The surgery thus far should involve little blood loss; the major hazard is at the junction between the hard and soft palates where a large vessel crosses the midline, requiring careful use of the fine electrocautery knife; if the knife is too hot, the vessel will rupture. The presphenoid bone will now be visible and two features are important to identify: the suture between the presphenoid and basisphenoid bones, which overlies the ventral surface of the hypothalamus medial to the supraoptic nucleus, and the venous sinus in the basisphenoid bone, which overlies the neural stalk of the pituitary gland (Figure 1G–I).

Transpharyngeal surgery is commonly undertaken to expose the supraoptic nucleus of the hypothalamus, which lies on the surface of the hypothalamus immediately lateral to the optic chiasm. To expose the supraoptic nucleus, the lateral wing of the palatine bone and medial pterygoid plate are cleared of muscle using cautery, taking care not to cauterise close to the surface of the hypothalamus. The exposed bone is then drilled away to expose the trigeminal nerve bundle (Figure 1I,J). The nerve is removed from above the supraoptic nucleus using two pairs of fine forceps, one to tease apart and lift a portion of the nerve bundle at its visible rostral extent and the other to peel each portion of the nerve bundle away to be cut at its caudal extent. Removing this nerve leaves the supraoptic nucleus obscured by a large vein that runs along the lateral margin of the sphenoid bones (Figure 1K). This vein is compressed with pledgets of paper tissue that are wedged against the sphenoid bones rostrally and caudally; the pledgets must compact the vein into the sphenoid bones and must not compress the brain, and care must be taken to leave the artery that runs parallel and lateral to the vein intact. The dense vasculature of the supraoptic nucleus can be seen through the meninges. The best position for recording is as close to the bifurcation of the communicating artery and the middle cerebral artery next to the optic chiasm.

Because the supraoptic nucleus is densely vascularised, the brain will move with each pulse wave of blood flow, making it impossible to maintain a stable recording from the supraoptic nucleus if the meninges are removed unless other steps are taken to stabilise the recording site. For example, Dreifuss and Kelly, after removing the entire sphenoid bone, to exposing the supraoptic nucleus and neural stalk, covered it with 5% agar to limit the pulsations.^8^ A fine needle is used to make a small (<1 mm) incision through the meninges to allow the recording electrode access to the supraoptic nucleus (Figure 1k). However, if a microdialysis probe is to be placed on the surface of the brain for direct administration of drugs into, and/or extracellular fluid sampling from, the supraoptic nucleus,^17^ the meninges should be removed to allow free diffusion between the dialysate and parenchyma; the gentle pressure of the microdialysis probe on the surface of the supraoptic nucleus will dampen the pulsing of the brain with each heartbeat. The best microdialysis probe for this purpose is an in‐house designed U‐shaped microdialysis probe,^18^ bent to position the loop of the membrane flat onto the exposed supraoptic nucleus, with the recording microelectrode placed through the centre of the dialysis membrane loop.^17^

If transpharyngeal surgery is used to expose other ventral brain areas,^19^, ^20^, ^21^ the exposure can be extended rostrally, caudally, medially, and laterally using cautery for soft tissue and drilling for bone. Care must be taken not to disturb the artery that runs parallel to the sphenoid bone when extending the exposure rostrally, caudally, or laterally. Similarly, the communicating artery, middle cerebral artery, and the small artery that crosses the optic chiasm must not be damaged when removing the sphenoid bones medially.

It is possible to place a recording electrode into the supraoptic nucleus with a high degree of confidence, and supraoptic neurones can be identified by their distinctive activity patterns.^22^ Nevertheless, the gold standard for identification of supraoptic neurones remains stimulation of antidromic action potentials from their axonal projections to the posterior pituitary gland, which requires the placement of a stimulating electrode on the axons.^5^ The axons bundle together at the neural stalk of the pituitary gland en route to their terminal fields in the posterior pituitary, making this the ideal site for antidromic stimulation. The neural stalk lies on the midline under the basisphenoid bone and so further surgery is required to access it.

**FIGURE 2:**
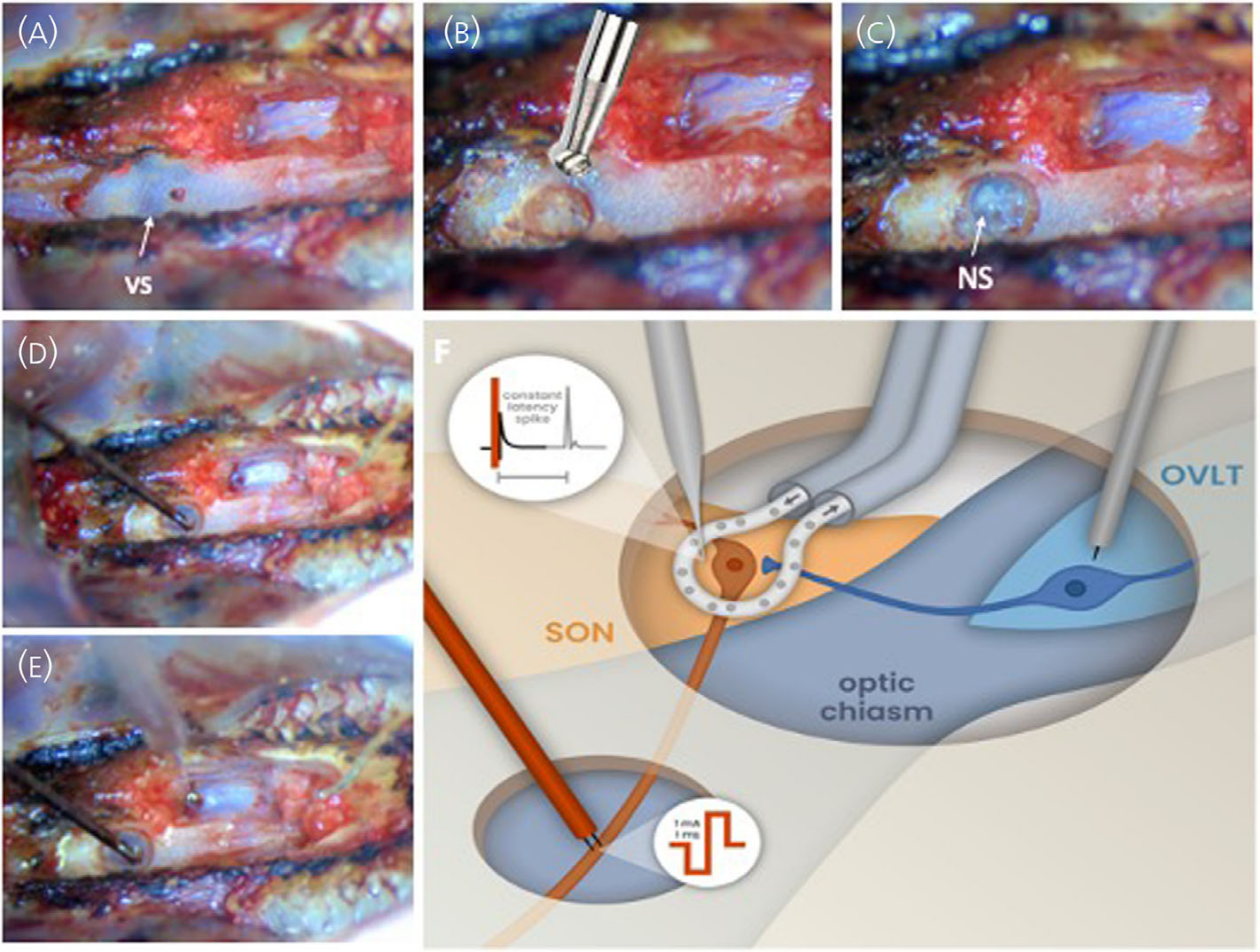
Step‐by‐step guide to transpharyngeal exposure of the neural stalk. (A) Above the neural stalk, the venous sinus within the basisphenoid bone can be seen at the rostral end of the pharynx. A shallow hole is drilled at the midline 2 mm caudal to the sinus and packed with bone wax. The hole is then progressively extended rostrally to push the bone wax ahead of the drill burr. (B) As the drilling breaks into the sinus, the drill spins bone wax into the sinus to prevent blood loss. (C) The hole is then drilled deeper to expose the rostral adenohypophysis and the neural stalk (NS), with the last thin layer of bone removed with fine forceps and a bent syringe needle. (D) The stimulating electrode is lowered onto the neural stalk and pushed few hundred μm into the tissue. (E) The recording electrode is lowered into the supraoptic nucleus using a micromanipulator. (F) Schematic representation of the full setup including a U‐shaped microdialysis probe with the recording microelectrode placed through the centre of the dialysis membrane loop and a second stimulating electrode placed into the OVLT. NS, neural stalk; OVLT, organum vasculosum of the lamina terminalis; SON, supraoptic nucleus; vs, venous sinus.

To expose the neural stalk, the venous sinus within the basisphenoid bone can be identified as a purple “smudge” at the rostral end of the pharynx (Figure 2A). The sinus can be the source of severe blood loss. To prevent blood loss, a shallow hole is drilled at the midline 2 mm caudal to the sinus and packed with bone wax. The hole is then progressively extended rostrally to push the bone wax ahead of the drill burr and into the sinus when reached; the bone wax must be replenished at regular intervals (Figure 2B). As the drilling breaks into the sinus, the drill spins bone wax into the sinus to prevent blood loss. The hole is then drilled deeper to expose the rostral adenohypophysis and the neural stalk (Figure 2C), with the last thin layer of bone removed with watchmaker's forceps and a tiny hook formed by the bent tip of a syringe needle. The exposure must be kept to the midline because extending it laterally engages blood vessels. The neural stalk should be visible through the dura: blood vessels converge at this site, giving an appearance that contrasts strongly with the pink tissue of the pituitary and the grey tissue of the median eminence rostral to the stalk. With practice, transpharyngeal surgery can be completed in ~30 min, with a loss of <0.5 mL of blood, but much larger amounts of blood can be lost from the basisphenoid sinus or the junction between hard and soft palates if they are not dealt with effectively.

Similarly to the supraoptic nucleus exposure, the neural stalk exposure can be extended to expand access. Extending rostrally will expose the median eminence, which also allows insertion of an injection cannula in the third ventricle. In addition, the visible sphenoid bones can be removed entirely, or “shaved” to a thin plate by drilling to allow access for a microscope objective for imaging. Even when the sphenoid bones are to be removed, the sinus must first be packed with bone wax to prevent excessive blood loss.

While transpharyngeal surgery provides a stable long‐lasting platform for in vivo studies, it should be noted that blood pressure after surgery is normally only 60–80 mm Hg.^23^

## 5 COMBINATION OF ELECTROPHYSIOLOGICAL RECORDING WITH MICRODIALYSIS

The ability of magnocellular neurones to release vasopressin and oxytocin from their whole membrane surface, and the fact that neuropeptides can diffuse through the extracellular space, has predisposed these neuropeptides to specialized sampling techniques such as in vivo microdialysis. The combination of electrophysiology recording with concurrent microdialysis allows local neurochemical dynamics to be related to the firing activity of identified neurones (Figure 2F). Furthermore, using microdialysis for local drug administration (retrodialysis) adjacent to an extracellular recording electrode provides for controlled introduction of neuroactive substances (e.g., receptor agonists and antagonists) to mimic or block endogenous neurotransmitter actions to modulate electrical activity in vivo.^17^ This can be further extended by placing stimulating electrodes to evoke trans‐synaptic inhibition or excitation from brain regions know to project to the supraoptic nucleus.^19^ Thus, the effects of input stimulation on local transmitter release and firing patterns can be studied in a single experiment to further our understanding of the mechanisms, dynamics and consequences of dendritic vasopressin and oxytocin release.^24^, ^25^

## 6 RECENT ADVANCES USING THE TRANSPHARYNGEAL APPROACH

While transpharyngeal surgery in rats has been used most extensively to study regulation of vasopressin and oxytocin neurone activity, a more extensive exposure than that described above enabled access for two‐photon imaging of the ventral surface of the brain. In this preparation, the right molars, the ventromedial aspect of the right temporal bone, the basisphenoid bone and the presphenoid bones were removed to expose the ventral surface of the hypothalamus and ventral aspect of the right temporal lobe. The first use of this more extensive exposure was pivotal in uncovering the role of supraoptic nucleus haemodynamics in the control of vasopressin neurone activity; hyperosmotic stimuli induce local vasoconstriction within the supraoptic nucleus creating local hypoxia that, in turn, increases vasopressin neurone activity.^26^ This was the first demonstration of “physiological” inverse neurovascular coupling, which had previously been thought to occur only in pathological situations. The assumption that neurovascular coupling matches energy supply to metabolic demand in healthy individuals does not apply to all brain regions. Hence, caution might be required in the interpretation of blood‐oxygen‐level dependent imaging and functional magnetic resonance imaging of deep brain structures.

This more extensive preparation was subsequently used to show that blood flows unidirectionally in a previously unknown portal blood system that likely delivers vasopressin from the suprachiasmatic nucleus to the organum vasculosum of the lamina terminalis (OVLT), and that the flow rate through the portal vessels is highest at night.^27^ The OVLT is a hub for the regulation of a variety of diurnally regulated physiological functions (including body fluid balance) and behaviours, so this newly identified portal system might be central to circadian regulation of biology.

## 7 CONCLUDING REMARKS

Transpharyngeal surgery has been a staple of neuroendocrine research for half a century. As new technology develops that can be utilised in this preparation, long‐standing questions can be addressed, and new questions can be formulated. Electrophysiological recording from the supraoptic nucleus has been limited to single‐unit recording using micropipettes. This has limited the number of neurones that can be concurrently recorded from an individual, either by placing two micropipettes (Belin et al., 1984) or by discrimination of units when two active neurones are serendipitously recorded through a single micropipette.^28^, ^29^ With the advent of recording systems that can concurrently record many neurones (e.g., neuropixels), it is now possible to determine the level of co‐ordination across the population of supraoptic nucleus neurones, which has long been speculated upon without the technology to challenge the hypotheses.^30^, ^31^

As described above, it is now possible to visualise the ventral surface of the brain with two‐photon imaging. The expanded exposure required for such imaging opens a much larger area of the ventral surface of the brain than previously thought possible. As such, the entire ventral surface of the hypothalamus and the ventral aspect of the right temporal lobe are now accessible in this preparation. The corresponding authors welcome approaches for advice in using this preparation for future experiments.

## AUTHOR CONTRIBUTIONS

Conceptualization, M.L., C.H.B. and G.L; Writing – Original Draft, M.L., C.H.B. and G.L.; Resources and Funding Acquisition, G.L, and M.L; All authors have approved the final version of the manuscript and agree to be accountable for all aspects of the work. All persons designated as authors qualify for authorship, and all those who qualify for authorship are listed.

## FUNDING INFORMATION

This work was supported by a grant from the Biotechnology and Biological Research Council (BB/S021035/1) (G.L., D.M., M.L.).

## CONFLICT OF INTEREST STATEMENT

The authors declare no conflicts of interest.

## Data Availability

Data sharing not applicable to this article as no datasets were generated or analysed during the current study.
